# Effects of Low Concentrations of Docosahexaenoic Acid on the Structure and Phase Behavior of Model Lipid Membranes

**DOI:** 10.3390/membranes5040857

**Published:** 2015-12-04

**Authors:** Chai Lor, Linda S. Hirst

**Affiliations:** 1 Bioengineering and Small Scale Technologies, School of Engineering, University of California, Merced, CA 95343, USA; E-Mail: clor@ucmerced.edu; 2 Department of Physics, School of Natural Sciences, University of California, Merced, CA 95343, USA

**Keywords:** DHA, lipid bilayer, gel phase, X-ray diffraction

## Abstract

In this paper we report an X-ray diffraction study on the phase behavior of binary lipid mixtures of 1-palmitoyl-2-docosahexaenoyl-*sn*-glycero-3-phosphoethanolamine (DHA-PE) and 1,2-dipalmitoyl-*sn*-glycero-3-phosphocholine (DPPC) at low concentrations below 5.0 mol% DHA-PE. Our results show that DHA-PE induces phase separation into a DHA rich liquid crystalline (L_α_) phase and a DHA poor gel (L_β′_) phase at overall DHA-PE concentrations as low as 0.1 mol%. In addition, we find that the structure of the L_β′_ phase, from which the DHA-PE molecules are largely excluded, is modified in the phase-separated state at low DHA-PE concentrations, with a decrease in bilayer thickness of 1.34 nm for 0.1 mol% at room temperature, compared to pure DPPC bilayers. This result is contrary to that seen in similar studies on mono-unsaturated lipids where an increase in bilayer thickness is observed. The surprising effect of such low DHA-PE concentrations on membrane structure may be important in understanding the role of highly polyunsaturated lipids in biological membrane-based structures and similar artificial surfactant systems.

## 1. Introduction

Since early epidemiological studies of the Eskimo diet linked docosahexaenoic acid (DHA) content [1] to cardiac health, polyunsaturated fatty acids have attracted a great deal of attention in the physiological, nutritional and biomedical communities. Numerous studies have drawn links between the high dietary DHA content of diets rich in fish oil to beneficial effects on cardiovascular disease [2], various cancers [3,4], arthritis [5], depression [6], Alzheimer’s disease [7], its role in infant development [8,9], and in retinal cell function [10]. Claims focused on the dietary benefits polyunsaturated fatty acids have led to a rapid expansion in the incorporation of DHA in the human diet from the beginning of infancy to senior adults. Studies have also shown that excessive intake of DHA can be dangerous, for example, in postnatal growth retardation [11]. Despite intense interest in these ubiquitous molecules there is a significant gap in our knowledge of the physiological effects of DHA-lipids and other polyunsaturated lipids in the diet and our current understanding of its specific biomolecular importance.

Certain classes of biological cells contain high concentrations of polyunsaturated lipids in the membrane. For example, DHA-lipids are particularly enriched in the synapses of neural membranes [12] and in the rod-cell outer segment in the retina [13]. In these special cases, the amount of DHA present can approach almost 50% of the total membrane fatty acids and di-DHA phospholipids are present [14,15]; in which both phospholipid chains on the same molecule have the characteristic 22:6 (ω3) DHA composition (alkyl chains 22 carbons long with 6 unsaturated bonds). DHA-lipid levels in other biological membranes can vary and are typically about 5%, although this content may be enriched by changes in diet [16]. In membranes altered by dietary DHA, it is possible that di-DHA phospholipids exist in significant amounts and account for the reported DHA-induced alterations in membrane function.

The effects of polyunsaturated lipids on membranes most likely originate in structural changes induced by the incorporated molecule. Polyunsaturated lipids may differ in length significantly to the host lipid molecules of a membrane since the unsaturated bonds allow the lipid to exhibit a large number of potential conformations, reducing the average length of the molecule. The most common arrangement for the alkyl chains in phospholipids is to have a saturated chain fatty acid, such as palmitic or stearic acid in the *sn-1* position and an unsaturated fatty acid such as oleic, linoleic or docosahexaenoic acid in the *sn-2* position [17,18,19]. We focus on DHA-lipid since its biological relevance is high, non-esterified DHA has been linked to some physiological functions but evidence for *in vivo* effects is lacking [20].

Despite significant nutritional and biomedical research, the fundamental physical significance of DHA-lipids in the plasma membrane and their interactions with other membrane lipids are still not fully understood and this is the reason why we use to a simple lipid membrane binary model. It is particularly interesting to look at the effects of DHA-lipid on a saturated membrane as such a system will likely exhibit phase separation.

Model membrane studies have changed our view of the fluid mosaic model [21] for biological membranes in demonstrating that lipid domains can arise and may play an important role in facilitating cellular functions at the membrane level. Lipid domains are in-plane lipid patches in the membrane that have a different lipid composition to their surroundings. In understanding the basic phase behavior of lipid molecules in a membrane, several recent studies have examined interactions between lipids from different categories; saturated, polyunsaturated, and sterols. The phase behavior of binary examples such as 1,2-dipalmitoyl-*sn*-glycero-3-phosphocholine (DPPC)/cholesterol [22,23], 1,2-dioleoyl-*sn*-glycero-3-phosphocholine (DOPC)/cholesterol [24], and DPPC/DOPC [25] is now well known, mapping out the regions of the gel (L_β′_, also known as S_o_) and liquid disordered, or liquid crystalline (L_α_) phases. Expanding model systems into ternary mixtures and beyond brings us closer to modeling the plasma membrane, but with increasing complexity.

Despite the fact that there have been recent studies on DHA lipids in ternary bilayers, not much has been done with a binary system [26,27,28,29,30], an important starting point to gain a basic understanding of how DHA lipids interact with saturated lipids. This article presents a detailed look at the low concentration regime of 1-palmitoyl-2-docohexaenoyl-*sn*-glycero-3-phosphoethanolamine (DHA-PE) in the DHA-PE/DPPC binary system using X-ray diffraction. DHA’s interaction with other lipids in model systems was previously studied at relatively high concentrations by Stillwell and Wassall [30,31,32]. In these studies, phase separation was indicated in mixtures of DHA and sphingomyelin at 10 mol% DHA-lipid using differential scanning calorimetry [31]. In another binary mixture of just cholesterol and DHA-lipid, it was shown that cholesterol has a very low solubility in DHA because of highly disordered chain packing in the system [32]. In the combined ternary mixture of DHA-lipid, cholesterol, and sphingomyelin, they also found that cholesterol has a greater affinity for the more rigid sphingomyelin causing the separation of the membrane into a DHA-rich/chol-poor and chol-rich/DHA-poor domains [30]. DHA has also been shown to induce morphological changes in the form of spherical or tubular structures when added into supported lipid bilayer using quartz crystal microbalance [33,34].

In general, DHA is a challenging lipid molecule to work with due to its high susceptibility to peroxidation. DHA has a total of 6 double bonds that lie in between methylene bridges, therefore the hydrogens in the fatty acid chain are very reactive. Free radicals in the environment can strip these hydrogens from the lipid thus turning that particular region into a radical itself until it becomes stable. The end products of lipid peroxidation are malondialdehydes [35]. There are several methods that can reduce or prevent oxidation of lipids such as the use of antioxidants, limiting light, and an oxygen free environment. Antioxidants such as α-tocopherol (vitamin E) can inhibit the oxidation process because it actively scavenges for reactive oxygen species. In measuring the rate of lipid peroxidation, the thiobarbituric acid assay (TBA) is commonly used. TBA reacts with malondialdehydes resulting in fluorescence emission that can be detected by spectroscopy. The TBA assay has been used in many research studies to measure lipid oxidation in cells [36,37]. One drawback of this technique is that it is not only specific to lipid as there are other methods to form malondialdehydes. However, in our research, it is sufficient because our mixtures contain only lipids.

X-ray diffraction is an excellent technique for characterizing lipid membranes and in this paper, we present data for lipid membranes prepared from the binary mixture DPPC/DHA-PE. Small angle X-ray scattering (SAXS) is used to probe length scales between 2 and 100 nm, producing an ensemble measurement representative of thousands of stacked bilayers. Our data provides information on the bilayer thickness and electron density distribution in different co-existing lipid phases. Wide-angle X-ray scattering (WAXS) was also carried out to look at shorter length scales. This technique reveals additional information on lipid in-plane organization within the bilayer.

It is clear that DHA is a biologically important fatty acid present in all of our cells and this molecule can have a significant impact on both specialized and general cell function. Fundamental research into the physical interactions between DHA containing lipids and the other lipids in the cell membrane however is still needed, particularly in relation to phase separation effects and their relevance to lipid domain formation. In this work we focus on the lower concentration limit of DHA membrane content and the effects of this molecule on membrane phase behavior in this regime.

## 2. Experimental Section

**Figure 1:**
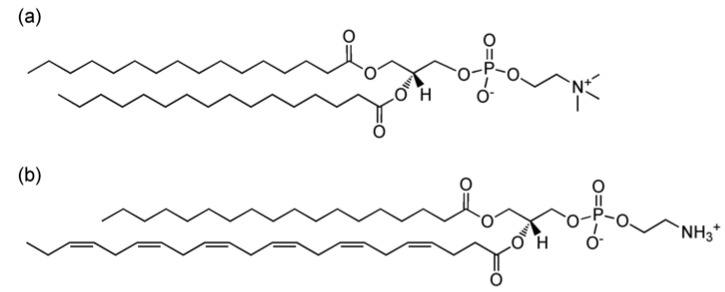
Molecular structures for the lipids used in this study: (**a**) 1,2-dipalmitoyl-*sn*-glycero-3-phosphocholine (DPPC); (**b**) 1-palmitoyl-2-docosahexaenoyl-*sn*-glycero-3-phosphoethanolamine (DHA-PE).

1-palmitoyl-2-docohexaenoyl-*sn*-glycero-3-phosphoethanolamine (DHA-PE) and 1,2-dipalmitoyl-*sn*-glycero-3-phosphocholine (DPPC) were obtained from Avanti Polar Lipids (Alabaster, AL, USA) in chloroform and used without further purification. α-tocopherol was obtained from Sigma-Aldrich (St Louis, MO, USA) and prepared as a stock solution of 1mM in chloroform. Figure 1 shows molecular structures for the lipids used in this study. All lipids were initially stored under −20 °C in chloroform. To prepare multilamellar vesicles for SAXS experiments, DHA-PE and DPPC were mixed by mol% in chloroform with 0.5 mol% α-tocopherol (an antioxidant) to a final lipid concentration of 25 mM. Mixtures were then dried with high purity nitrogen and vacuum dried for 2 h to further remove any excess chloroform. Samples were then rehydrated with Millipore water back to 25 mM and incubated at 45 °C from 5–10 min to release the lipid from the vial’s glass wall. The samples were finally vortexed to ensure that the lipids were well dispersed with a milky white appearance. To prepare X-ray samples, these solutions are pipetted into 1.5 mm glass capillaries and centrifuged at low speed to produce pellets in the fully hydrated lamellar phase. Furthermore, the capillaries were sealed with silicon rubber sealant immediately after filling to prevent any evaporation of the water and limit exposure to oxygen. Because DHA is highly susceptible to lipid oxidation, sample preparations were carried out rapidly under limited light conditions and under high purity nitrogen. Capillaries were then stored at 4 °C until the X-ray measurements were carried out.

α-tocopherol was used as an antioxidant to prevent oxidation of DHA. We used a TBA assay to measure the degree of malonaldehydes produced from lipid peroxidation [38]. Concentrations of 0.5 mol%, 1.0 mol%, and 2.0 mol% α-tocopherol were added to 1 mM DHA solutions against a control of no α-tocopherol. TBA was added to the solutions and incubated to create TBA reactive species (TBARs). We used the Nanodrop spectrophotometer to measure the absorbance of TBARs at 532 nm and observed that all three concentrations of α-tocopherol equally showed no significant oxidation vs the control where oxidation was clearly observed. (See Supplementary Materials, Figure S1).

SAXS measurements were carried out at the Stanford Synchrotron Radiation Lightsource (SSRL) beamline 4-2 at the Stanford Linear Accelerator Center (SLAC). We used a wavelength of 1.127 Å (11 keV) with a 1.7 m distance from sample to detector in the SAXS configuration. Powder diffraction patterns were recorded on a CCD detector (Rayonix MX225-HE) using a 1 s exposure time. For instrument calibration, we used silver behenate (*d_001_* = 58.83 Å) with a sensitivity of 1 Å. Capillaries were mounted into an aluminum temperature chamber designed specifically for this beamline. Using this apparatus, capillary temperature was controlled to an accuracy of ±0.25 K by passing water through channels in the chamber. Thermistors were mounted close to the sample to monitor the temperature. After collecting powder diffraction patterns using a CCD camera, SasTool software at the beamline was used for radial integration (producing plots of scattered intensity as a function of scattering vector, **q**). Origin Lab was used for peak finding and further analysis.

## 3. Results and Discussion

### 3.1. Phase Behavior of DPPC/DHA-PE Mixtures

**Figure 2:**
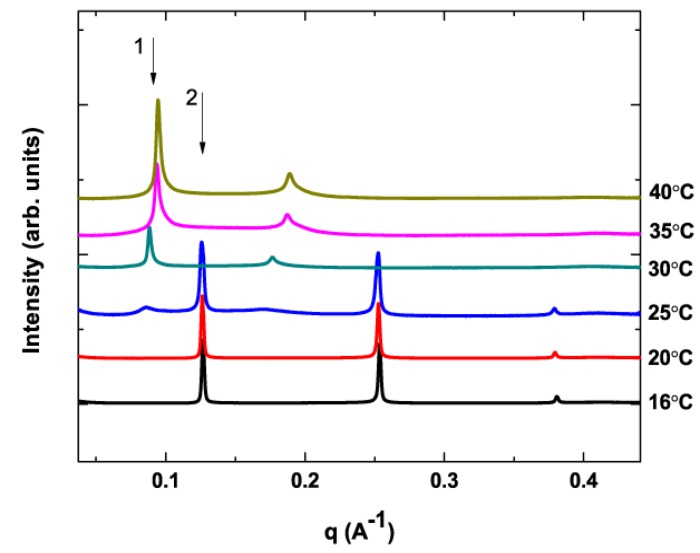
Transmission solution small angle X-ray scattering (SAXS) data for mixture of DPPC/DHA-PE at DHA-PE 0.1 mol%. At 25 °C, phase coexistence can be observed as two sets of Bragg peaks are present. The first order peaks for each phase are indicated by arrow 1 (fluid phase—L_α_ ) and arrow 2 (gel phase—L_β′_).

Pure DHA-PE has a gel to fluid phase transition (T_m_) at −27 °C in contrast to that of DPPC at 41 °C. The relative immiscibility of these lipids within a certain temperature range drives phase separation and we observe that even very small amounts of DHA-PE lipid significantly modify the phase behavior or the system. Lipid mixtures were prepared at different DPPC/DHA-PE ratios with 0.5 mol% α-tocopherol and SAXS/WAXS diffraction data collected as a function of temperature. Figure 2 shows an example of X-ray scattering data for 0.1 mol% DHA-PE with increasing temperature. The data are plotted as scattered intensity as a function of scattering vector, **q**, and Bragg peaks corresponding to scattering from stacks of bilayers can be seen. At lower temperatures the L_β′_ phase is clearly present, with 3 orders of diffraction shown from the lamellar stack at *q* = 2π/d, 4π/d, and 6π/d. The gel phase in lipid membranes can typically produce 5 orders of diffraction or more because of its long-range inter-bilayer ordering but here only 3 orders are shown as the detector was positioned to collect data up to *q*_max_ = 0.45 Å^−1^. This *q* range is sufficient to clearly differentiate the L_β′_ gel phase from the L_α_ fluid phase. The gel phase also exhibits a WAXS (Wide angle X-ray scattering) peak at ***q*** *=* 1.57 Å^−1^ corresponding to in-plane lipid packing and the presence of this peak can be used to confirm the gel phase. Based on this X-ray evidence we can unambiguously distinguish between the gel (L_β′_) and the fluid phase (L_α_) in our system.

Owing to distinct differences between the gel, fluid and liquid ordered (l_o_) phases in lipid mixtures in terms of molecular ordering and bilayer-bilayer stacking order, we previously demonstrated the use of SAXS to distinguish between scattering patterns for each of these phases and to confirm two and three-phase coexistence in ternary lipid mixtures [39] by observing distinct sets of Bragg peaks differing in peak width and number of orders present (representative also of membrane rigidity). In the case where bilayers exhibit phase separation, similar domains tend to stack together. This fact is evident both from the success of the technique itself (randomly arranged domains in the stack would not produce two sets of Bragg peaks) but was also confirmed independently by fluorescence microscopy on membrane stacks [40].

In Figure 2, the first order peak from the L_β′_ phase is seen at ***q*** = 0.126 Å^−1^ corresponding to a bilayer spacing of *d* = 4.99 nm (Figure 2). As the temperature was increased to 25 °C, we observe the emergence of a second peak at ***q*** = 0.897 Å^−1^ corresponding to *d* = 7.00 nm. This peak can be assigned to a stack of membranes in the L_α_ phase [38]. Unlike the L_β′_ phase, the L_α_ phase only produces 2 orders of diffraction at ***q*** = 0.897 Å^−1^ and ***q*** = 0.178 Å^−1^. This scattering signature, more characteristic of short range ordering in the bilayer stack is expected due to increased thermal membrane fluctuations in the more fluid L_α_ phase. The two peaks indicated by the arrows at 25 °C in Figure 2 are indicative of phase coexistence in the sample at that temperature. Above 30°C, the L_β′_ phase completely disappears as the membrane becomes homogenous at T_m_. We can also note at this point that the L_α_ phase has a bilayer d-spacing approximately 2 nm larger than that of the L_β′_ phase. The d-spacing of the L_α_ phase gradually decreases with increasing temperature to *d* *=* 6.65 nm at 40 °C. Such behavior is expected as increased thermal motion of the lipid tails results in an increase in the lateral distance between neighboring molecules and therefore a decrease in bilayer thickness [41,42].

### 3.2. No X-ray Beam Damage

In working with DHA-PE, we want to verify that the lipids sealed in the capillaries did not undergo any molecular damage due to either the X-ray beam during exposure, heating, or over the time of our experiment. To control for these external factors, data was collected when the thermal chamber reached the desired temperature and 30 min afterwards. Data collection at each temperature occurred within 1–1.5 h because it took longer for the chamber to stabilize at higher temperatures. The data at 40 °C was collected approximately 12 h after that at 20 °C. The sample clearly went into the fluid phase at 40 °C and then the sample was allowed to cool back to room temperature. After roughly 48 h, we recollected data for the same sample and found the results to be identical to the previous data at that same temperature (See Supplementary Materials Figure S2). These test experiments confirmed that the sealed lipids did not undergo any molecular modifications detectable in our experiment due to beam damage, heating, or with time over the timescales of our experiments.

### 3.3. Phase Diagram of DPPC/DHA-PE

**Figure 3:**
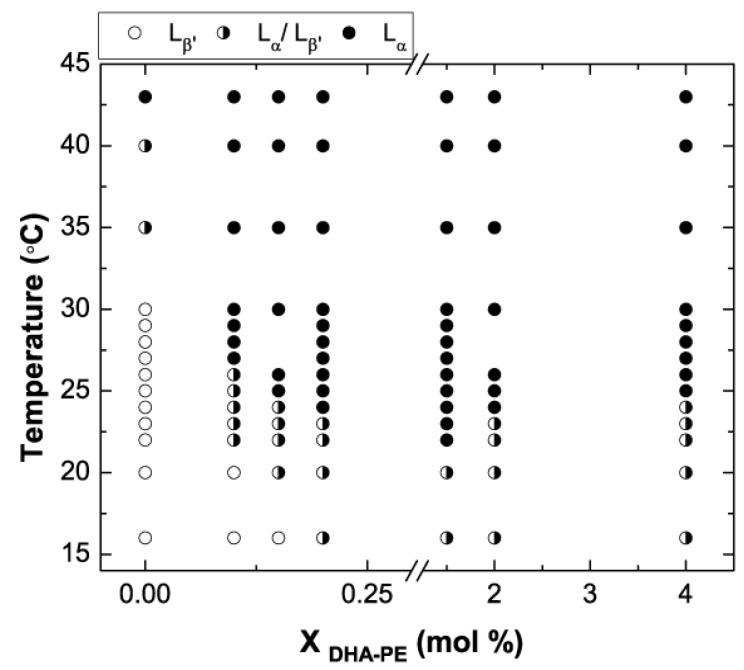
Binary phase diagram of DPPC/DHA-PE at low DHA-PE concentrations as determined by SAXS.

Figure 3 shows a phase diagram in which we map out the regions of L_β′_, L_α_, and phase coexistence from 16 to 43 °C using the above described X-ray analysis. 16 °C was the lowest achievable temperature for our experimental setup using chilled water to cool an aluminum chamber. The system can be heated reliably to 45 °C but beyond this point it was difficult to maintain a stable temperature due to heat dissipation. Another limitation to temperature accuracy came from the size of the scattering window across the chamber, a significant source of heat loss. As a result we estimated the temperature at the sample to be accurate to ±2 °C. Future experimental work will involve an improved design for this chamber.

From the data set shown in Figure 3 we can observe some key points. Firstly, a full membrane in the gel phase is only present at lower temperatures with less than 0.2 mol% DHA-PE. We also notice that the phase boundaries with added DHA are a function of both composition and temperature. Above 1% DHA the transition temperature (T_m_) does appear to increase with added DHA. At the concentrations shown in the figure, a phase coexistence region spans approximately 7 °C before transitioning completely into the fluid phase at T_m_. In the 0 mol% case (Pure DPPC), scattering characteristic of the ripple phase was also observed with the expected temperature range [43]. Evidence of the ripple phase however was not seen in any of the other mixtures, although it is possible that it may be present with a narrow temperature range and was not detected by our experiment.

### 3.4. Electron Density Profile Calculations

**Figure 4:**
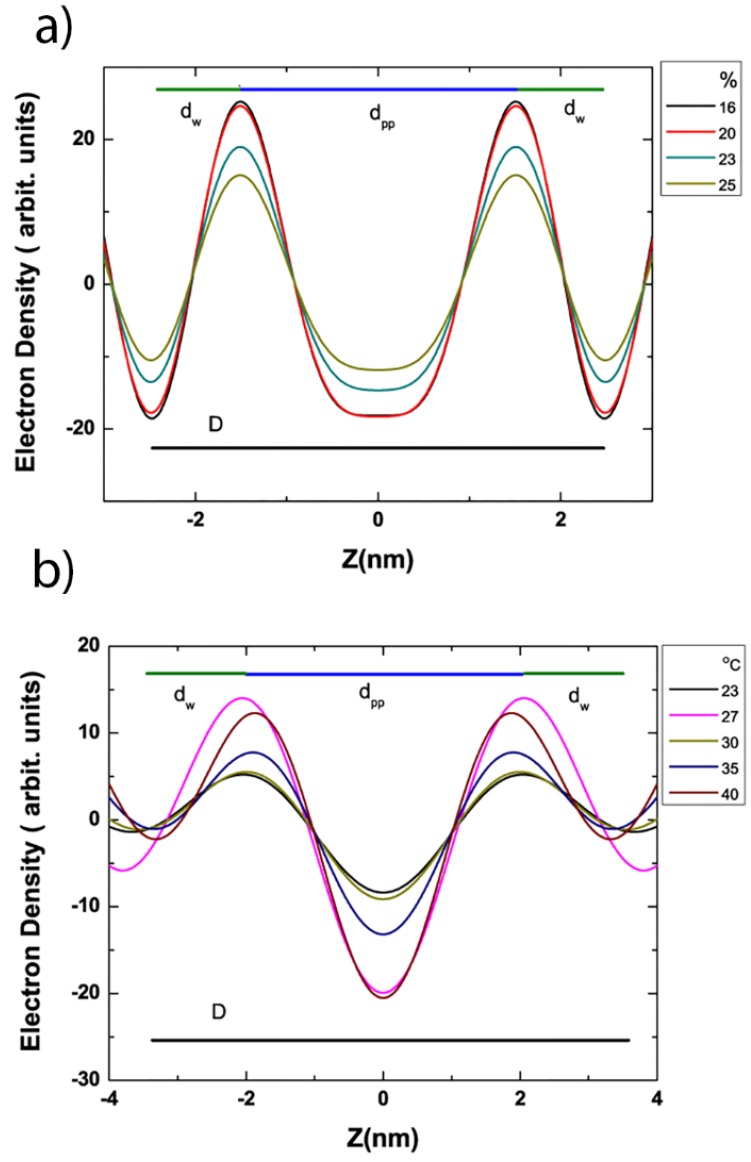
Electron density profiles for the coexisting (**a**) L_β′_ and (**b**) L_α_ phase at 0.1 mol% DHA-PE at different temperatures. The center of the bilayer is at *Z* = 0 nm.

From our SAXS data, it was possible reconstruct preliminary electron density profiles and analyze the structure of the bilayer in more detail for the L_α_ and L_β′_ phases. We used this method primarily to obtain an estimate for the average distance between lipid headgroups in the two leaflets (d_pp_) and to calculate the average thickness of the water layer (d_w_) for both phases, where d_w_ is the difference between the complete bilayer spacing, (d) and d_pp_. Our calculations followed Quinn and Wolf’s [44] method to create the electron density distribution graph as a function of thickness (Z) for our data as shown in Figure 4. In the bilayer, the phosphate head groups are expected to have the highest electron density due to close packing. The region where the hydrocarbon chains meet (Z= 0 nm) is expected to have the lowest density. Using this technique we looked at the electron density profiles of both the L_β′_ and the L_α_ phase coexisting in domain stacks within the bilayer stacks that make up the sample [37,38]. From our analysis, we obtained a d_pp_ thickness of 3.03 ± 0.06 nm using the 3 Bragg orders collected in the L_β′_ phase at 0.1 mol% and 20 °C.

In general, this calculation should be performed using 4 or more orders of diffraction. After a careful analysis of EDP reconstruction comparing the use of 3 orders with 4 and 5 orders for one mixture, we found that our calculation of d_pp_ would be expected to be too low with a 10% systematic error. While it was clear that performing the calculation using more orders of diffraction provides a more accurate estimation of the structure, unfortunately this was not generally possible as q_max_ for most of our data was too low. To account for this error we corrected our data accordingly to give an adjusted d_pp_ of 3.33 ± 0.06 nm. Using this method we can obtain a water layer of *d*_w_ = 1.66 ± 0.06 nm for the L_β′_ phase fraction in 0.1 mol% DHA-PE membranes as shown in Figure 4a.

The water layer from this analysis is quite close to the 1.83 nm water layer for pure DPPC L_β′_ phase in excess water from Torbet and Wiklins [45]. For the L_α_ phase fraction at 0.1 mol% DHA-PE, we calculated the d_pp_ and d_w_ to be 3.96 ± 0.05 nm and 3.35 ± 0.05 nm respectively. In the calculation for the liquid disordered phase, only 2 orders of diffraction are present, as is characteristic of the short range ordering in this phase therefore no correction to the EDP data was needed. Figure 4b shows that the volume occupied by the headgroup peak in the L_α_ phase is very broad, not as sharp as the L_β′_ phase and this is an indication that the phase exhibits high fluctuations, In other words, our calculation of the thickness of the water layer in the L_α_ phase is an approximation, since the region is less well defined compared to that of the L_β′_ phase.

From EDP analysis were able to make two key conclusions, firstly that the water layer, d_w_, associated with membranes in the L_β′_ phase for the mixtures we studied does not change significantly when compared to the expected value for pure DPPC and therefore subsequently that any differences we observe in bilayer d-spacings in this phase are a result of differences in d_pp_, the membrane thickness.

### 3.5. Bilayer d-Spacing Plots

**Figure 5:**
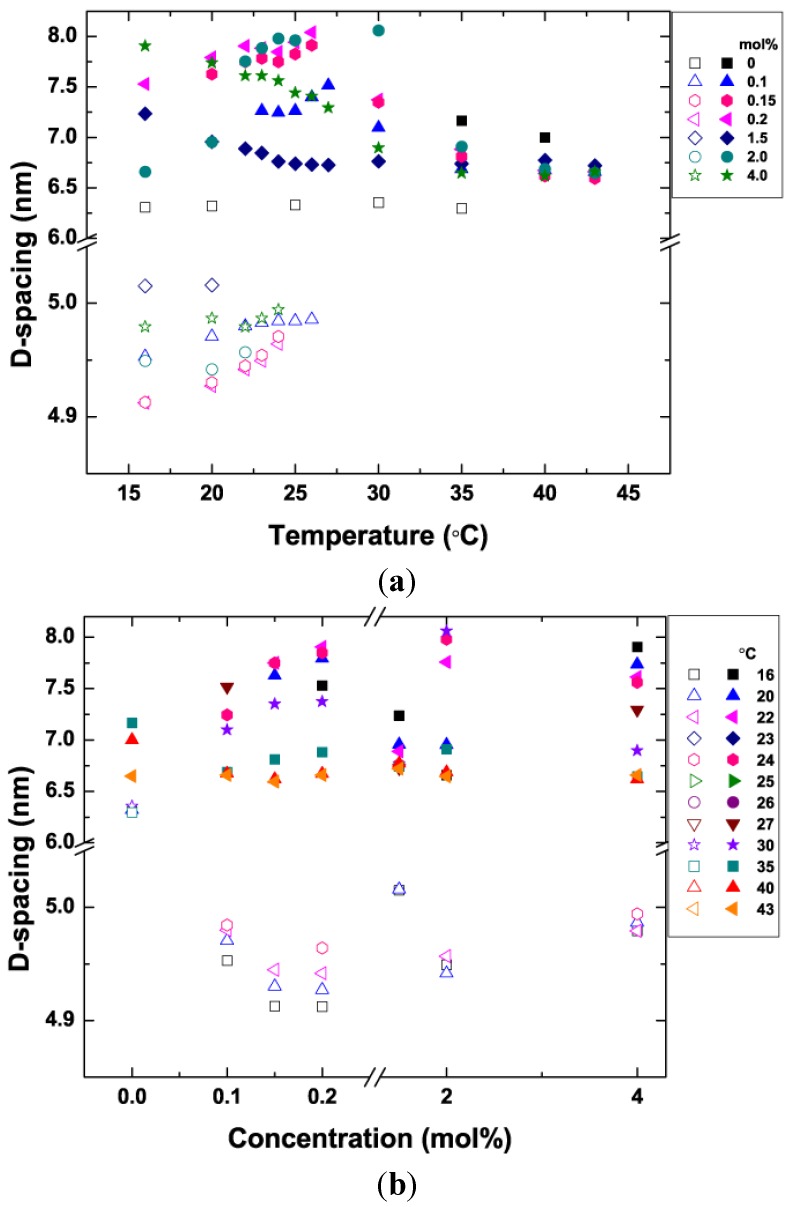
Lamellar d-spacing in the L_β′_ (open symbols) and L_α_ (solid symbols) phases as a function of: (**a**) temperature (for different DHA concentrations); and (**b**) DHA concentration (at different temperatures). Where the two phases coexist both open and solid symbols are present at the same temperature (plot a) or concentration (plot b).

From our SAXS data taken across a range of lipid mixtures, we plotted two graphs: bilayer d-spacing *vs.* temperature and d-spacing *vs.* concentration for both the L_β′_ and the L_α_ phases (Figure 5). In the temperature plot (Figure 5a), phase co-existence, where both L_β′_ and L_α_ are present is denoted by the presence of both open and solid symbols of the same shape for each concentration shown. As we saw in Figure 3, even at low temperatures the higher concentrations exhibit phase separation. Due to the presence of DHA. Note also that the square 0% points (pure DPPC) do not change significantly as a function of temperature. The L_β′_ phase completely disappears at 30 °C for all the concentrations that include some DHA-PE. From looking at the data, we can see the dependence of the L_β′_ phase d-spacing on temperature (open symbols). This small increase of approximately 0.05 nm is likely due to increased thermal fluctuations in the membrane with increasing temperature.

The membranes all exhibit homogeneous L_α_ phase (solid symbols) at 30 °C and above. As the temperature increases, the d-spacing of the L_α_ phase decreases slightly. This effect can be explained thermally. Heating causes an increase in the average lateral area occupied by each molecule, providing a larger volume for the highly flexible DHA chain to occupy and allowing for a slightly thinner bilayer. In Figure 5b, we use the same data set to plot d-spacing for both phases as a function of DHA-PE concentration. Results for different temperatures are shown using different symbols. From this plot we can more easily observe that an increase in the concentration of DHA-PE doesn’t have a significant effect on the d-spacing of either phase when phase separated at fixed temperature.

At the very low concentration of 0.1 mol% DHA-PE, we were able to observe phase separation into L_β′_ and L_α_ phases at room temperature (22 °C). Pure DPPC membranes have T_m_ at 43 °C and at room temperature exhibit the tilted L_β′_ phase with a d-spacing of 6.4 nm [46]. Our results demonstrate that the addition of a very small amount of DHA-lipid to the system as a whole decreases the bilayer spacing of this phase fraction dramatically, despite the fact that we expect most of the DHA-PE molecules to partition into the L_α_ phase. Previous studies of binary systems that also include DPPC such as, DPPC/DOPC [47] and DPPC/cholesterol [20,45] have shown that the tilt of the L_β′_ phase reduces to 0° on addition of different lipids accompanied by a decrease in membrane rigidity as shown in an AFM study of DPPC/cholesterol [48]. These effects cause the bilayer thickness in the L_β′_ phase to increase. We initially assumed that the DHA molecule would perform the same way as cholesterol and DOPC by alleviating the tilted gel phase due to its high degree of unsaturation. However, instead of increasing the thickness of the L_β′_ phase, we observed that membrane thickness in the L_β′_ phase was decreased a significant amount from that measured in pure DPPC L_β′_ phase, both at low temperatures where a single L_β′_ phase is present and also in the phase separated state.

### 3.6. Proposed Cause of Decreased Bilayer Thickness in L_*β*′_

**Figure 6:**
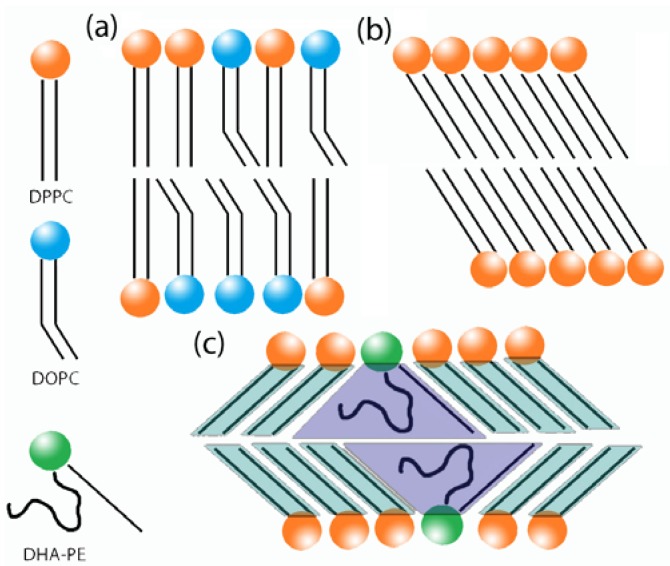
Schematic of the L_β′_ phase in pure: (**a**) DPPC/DOPC; (**b**) DPPC, and (**c**) DPPC/DHA-PE bilayers. DHA may locally induce increased tilt in the gel phase domains.

To fully understand the role of DHA in modifying the L_β′_ phase structure it is important to know the exact composition of the phase. This is unfortunately unknown in the phase-separated state, but we can assume a DHA-PE concentration is lower than 0.1 mol% DHA-PE using tie-line theory, since the exact lower phase boundary has not been determined. Figure 6 shows a schematic of the effect on tilt angle by incorporation of DOPC and DHA into DPPC L_β′_ phase.

To explain the dramatic change in d-spacing, we propose that instead of the DHA molecules reducing the rigidity of the L_β′_ phase, the molecule actually acts to increase the tilt angle of the L_β′_ phase. There are two possible scenarios that can potentially explain this phenomenon. In the first, DHA has a long hydrocarbon chain of 22 carbons compared to the 16 carbons of DPPC. The 22 carbon chain may be stretched out within the L_β′_ phase geometry because its freedom is being restricted by the DPPC rigid environment. The stretched unsaturated chain will cause the gap between the two leaflets to increase, therefore, the surrounding lipids will have to increase their tilt angle in order to reduce the gap and avoid a vacuum. In the second scenario, The DHA lipids are not extended. Due to the high number of possible configurations for DHA, on average filling out a volume space like a cone, steric interactions increase the tilt of the L_β′_ phase (Figure 6c). Entropically, this second case is the most likely since the DHA-lipid chain is not extended and there are many possible fatty acid conformations in this geometry. Moreover, it is possible that the interaction of DHA-PE can induce a cross-tilted gel phase causing the bilayer to be thinner as shown for the gel phase of DPPE in a molecular dynamics simulation by Leekumjorn [49].

#### 3.6.1. Increase in Tilt Angle

As mentioned above, the tilt angle of the gel phase is reduced to 0° when either cholesterol or DOPC is incorporated as shown by Schmidt [40] and Kamarker (Figure 6a) [21,46]. The L_β′_ phase in our system exhibits a dramatic bilayer thickness reduction from 6.28 nm in fully hydrated pure DPPC to 4.99 nm with 0.1 mol% DHA-PE at 22 °C for example. We suggest that DHA-PE changes the bilayer thickness by increasing the tilt of the L_β′_ phase. For a fully hydrated DPPC bilayer of d-spacing at 6.4 nm with a 1.83 nm water layer, the tilt angle is expected to be 32.6° as calculated by Katsaras [50]. By extrapolation, using our decreased d-spacing of 4.99 nm, d_pp_ = 3.33 nm and d_w_ = 1.66 nm, the new tilt angle would need to be 50.85° for a pure DPPC L_β′_ phase. This simple calculation assumes using a pure DPPC L_β′_ phase because the concentration of DHA-PE in the gel phase is very low. Such a high tilt angle in the membrane is unlikely and therefore we can expect other factors to play a role in the reduction in d-spacing.

To confirm the role of increased molecular tilt on bilayer thickness, wide angle X-ray scattering (WAXS) was also carried out at SLAC. Due to the significant change in the gel phase d-spacing from SAXS measurements, we expected that the lateral packing of the lipids would also change. For pure DPPC bilayer at 20 °C, we observed both the d_20_ and the d_11_ peak. The peak d_20_ is at *q* = 1.486 Å^−1^ (*d* = 4.228 Å^−1^) corresponding to the in-plane chain packing of the lipids. This result is in agreement with the WAXS peak of gel phase from Nagle [51]. WAXS was carried out for DHA-PE concentrations from 0.25 to 2.50 mol% across a range of temperatures (16 °C–35 °C). At 20 °C, we obtained a q value of *q* = 1.576 ± 0.002 Å^−1^ (*d* = 3.972 Å ± 0.005) for concentrations of 0.25–0.75 mol%. For 1.00–2.50 mol%, the *q* value dropped to *q* = 1.537 ± 0.002 Å^−1^ (*d* = 4.086 Å ± 0.005). We plotted the concentration of DHA-PE *vs.* d-spacing of the d_20_ peak (Supplementary Materials Figure S3). At all DHA-PE concentrations measured in this WAXS experiment, we observed that the chain packing of the gel phase containing DHA-PE molecules is lower than for pure DPPC membranes. This result is in good agreement with our proposed model of increased tilt shown in Figure 6c. When the samples were heated to the fluid phase at above 30 °C, the characteristic WAXS gel peak disappeared.

#### 3.6.2. Interdigitation

Increasing the tilt angle of lipid molecules is one possible way to cause a decrease in thickness but there are few other possibilities. A bilayer can be interdigitated, where the hydrophobic region becomes denser and hydrocarbon chains between opposing monolayer pack next to each other [52]. Also, interdigitated membranes have typically only been observed in bilayers of saturated lipids because unsaturated lipids statistically have many conformations, reducing the probability of interdigitation. Most interdigitated membranes are achieved through molecular modification, solvent selection or applying an external force [52]. Based on this evidence we assume that interdigitation can play only a small role here.

#### 3.6.3. Change in Water Layer Thickness

Another possible explanation for the observed decrease in d-spacing thickness is a substantial decrease in the water layer between the stacked L_β′_ membranes. We initially postulated that the water layer of the L_β′_ phase with DHA-PE could have a dramatic water layer decrease due to the presence of DHA-PE. However, from our electron density profile (EDP) analysis, we find that the change in the water later is particularly significant, with a difference of just 0.13 nm from a pure DPPC L_β′_ phase to L_β′_ phase with DHA-PE. This EDP analysis suggests that thickness changes in the bilayer (d_pp_ decreases by 1.15 nm) have a significant effect on bilayer thickness.

#### 3.6.4. Presence of α-Tocopherol

One important discussion point concerns the presence of α-tocopherol in our systems—this might be suggested as the cause of the decreased bilayer thickness in the gel phase. The gel phase is not completely free of DHA-PE in that there is a small amount present in each phase, thus resulting in a decreased transition temperature. NMR studies have shown that α-tocopherol prefers to localize close to DHA-PE in a symbiotic pair feeding on reactive oxygen species while protecting the lipid from oxidation [53]. α-tocopherol molecules that localize around the DHA-PE in the gel phase however are expected to have no effect on the bilayer thickness. It was shown by Kamal *et al.* that α-tocopherol does fluidize the DPPC gel phase and change the transition temperature, but this effect is only seen at much higher concentrations than those in our study. In addition, they observed no significant change in bilayer thickness even at high concentrations of α-tocopherol [54]. We have also carried out a control experiment with 0.5 mol% of α-tocopherol in DPPC membrane and observed no difference between this and the pure DPPC membrane that was plotted in Figure 3 and Figure 5. At 0.5 mol% of α-tocopherol, there is no effect on the bilayer thickness and T_m_. There is the question that α-tocopherol may preferentially partition into the gel or fluid phase, influencing the structure by exhibiting higher local concentrations. However since such effects are manifested as the ripple phase in Kamal *et al.* [54] this does not seem to be the case as the X-ray signature of that phase is distinct.

#### 3.6.5. Influence from Oxidized DHA-PE

A second important discussion point is the effect of oxidized DHA-PE on the gel phase. A recent model from Wallgren on oxidized lipids in the membrane suggested that the oxidized acyl chain caused disruption of the lipid molecules surrounding it by extending the *sn-2* chain towards the polar head group or into the water region. This effect can increase the chain packing density, as seen in our WAXS data [55,56,57]. It is possible that this could be the cause of our observations, considering that we use easily oxidized lipids, however, we have taken measures to prevent oxidation. Nonetheless, it we find it a very interesting result that such small amounts of DHA-PE at 0.1 mol% can have a huge effect on the gel phase bilayer thickness.

The effects of DHA-PE on the L_β′_ phase are clear evidence that there is some small volume of DHA-lipid in the L_β′_ phase and that this fraction modifies the membrane structure significantly or that the effects of DHA molecules surrounding these domains are able to modify the domain bilayer structure. We have focused on a DHA lipid containing one DHA fatty acid in the *sn-2* position*,* while we still have a saturated tail in the *sn-1* position. With only one tail that is highly unsaturated, we can assume that if we were to have a mixture of a lipid consisting of two DHA tails (di-DHA), even more dramatic differences in phase behavior may result. Such studies will be the subject of future work and may reveal interesting results related to the role of di-DHA in biological membranes.

## 4. Conclusions

In this paper, we investigated the phase behavior of a DHA-lipid with DPPC in the low concentration regime. Remarkably, DHA-lipid concentrations as low as 0.1 mol% induced phase coexistence of the L_β′_ and L_α_ phases and a lower concentration bound to this phase separated region in the phase diagram was not found. The effect of DHA on chain packing in the L_β′_ was also unexpected and possible evidence for a dramatic increase in lipid tilt. This tilt effect can be explained by geometrical arguments, although other factors clearly must play a contributing role. DHA-lipids are extremely prevalent in biological systems; however their interactions with membrane lipids, in the dynamic cell are not well understood. Fundamental work on reduced systems, such as we present here, can help us to gain a better understanding of the biological role of these important molecules.

## Supplementary Files

Supplementary File 1
