# Supplementary material for: Effects of Low Concentrations of Docosahexaenoic Acid on the Structure and Phase Behavior of Model Lipid Membranes
Source: Membranes (Basel). 2015 Dec 4;5(4):857–74. doi: 10.3390/membranes5040857 (PMC4704016; doi:10.3390/membranes5040857)
Supplement: Supplementary File 1 [file membranes-05-00857-s001.pdf]

Supplementary Materials

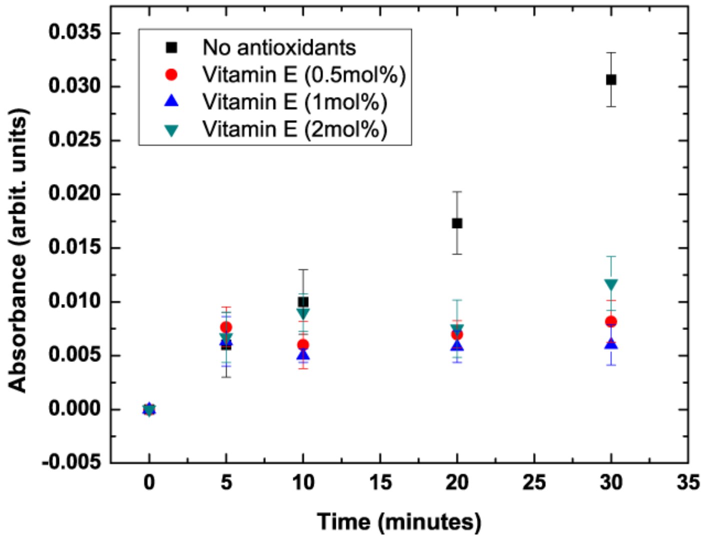

Figure S1. Oxidation quantification of DHA-PE in water over time with various amounts of ∝-tocopherol (Vitamin E) present. The thiobarbituric acid (TBA) assay is used to quantify the degree of oxidation by measuring the absorption of TBA reactive species at 532 nm. 0.5 mol% is sufficient to achieve minimal oxidation over the timescale of our experiments.

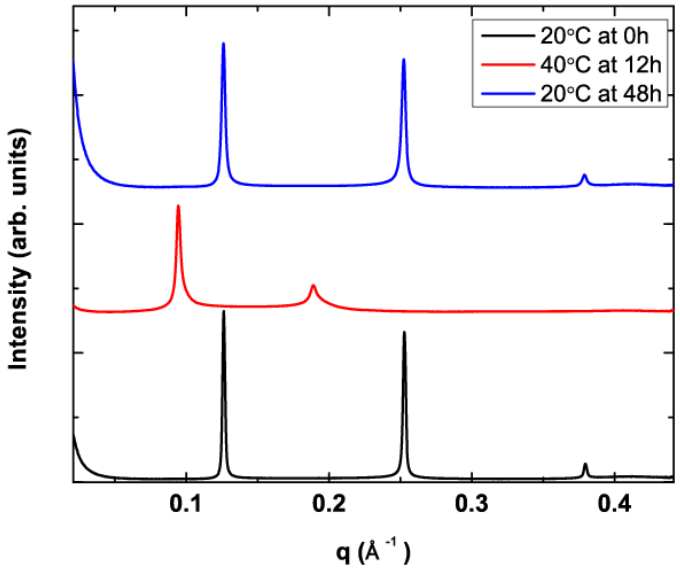

Figure S2. Small angle x-ray scattering of DHA-PE (0.10 mol%) in DPPC membrane. This data shows the heating and cooling of the membrane from gel phase to fluid phase over the course of 48 h. No significant changes in the scattering signature from the gel phase Bragg peaks are observed over this long timescale indicating that time dependent effects such as beam damage and oxidation do not affect membrane structure significantly.

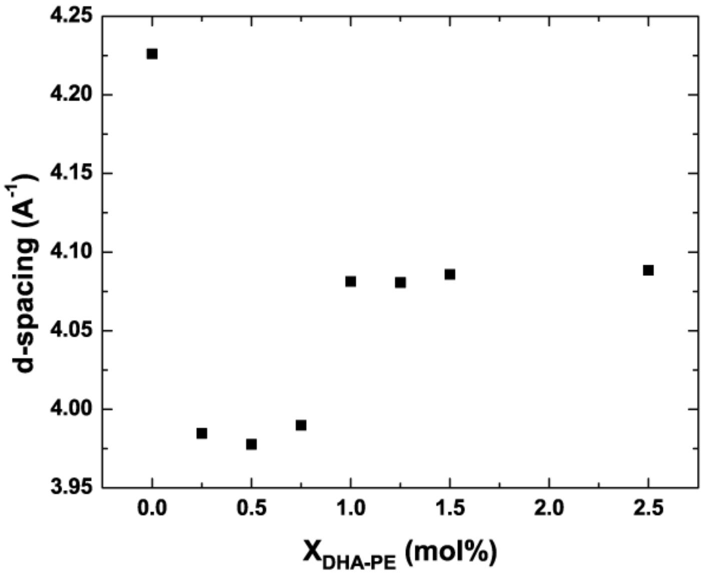

Figure S3. Plot of DHA-PE concentration *vs*. d-spacing from wide angle x-ray scattering (WAXS). The d-spacing shown here represents the lateral chain packing of lipid molecules.
